# The role of international partnerships in improving urethral reconstruction in low- and middle-income countries

**DOI:** 10.1007/s00345-019-02819-2

**Published:** 2019-06-08

**Authors:** Maahum Haider, Mohamed Jalloh, Jiaqi Yin, Amadou Diallo, Nancy Puttkammer, Serigne Gueye, Lamine Niang, Hunter Wessells, Kurt McCammon

**Affiliations:** 1 University of Washington, 1959 N.E. Pacific St, Box 356510, Seattle, WA 98195 USA; 2 IVUmed, Salt Lake City, USA; 3 Hopital General de Grande Yoff, Dakar, Senegal; 4 Eastern Virginia Medical School, Norfolk, USA

**Keywords:** Urethral stricture, Global surgery, International, Reconstruction, IVUmed

## Abstract

**Purpose:**

To explore the impact of education and training in international surgical partnerships on outcomes of urethral stricture disease in low- and middle-income countries. To encourage data collection and outcomes assessments to promote evidence-based and safe surgical care.

**Methods:**

Qualitative data were collected through observation of a reconstructive surgical workshop held by IVUmed at a host site in Dakar, Senegal. Quantitative data were collected through a retrospective review of 11 years of hospital data to assess surgical outcomes of urethral stricture disease before and after IVUmed started reconstructive workshops at the site.

**Results:**

In the 11-year study period, 569 patients underwent 774 surgical procedures for urethral strictures. The numbers and types of urethroplasty techniques increased after IVUmed started its workshops. The average number of urethroplasties increased from 10 to 18.75/year. There was a statistically significant improvement in the mean success rate of urethroplasties from 12.7% before to 29% after the workshops. Anastomotic urethroplasty success rates doubled from 16.7 to 35.1%, but this was not statistically significant (*p* = 0.07). The improved success rate was sustained in cases performed without an IVUmed provider.

**Conclusions:**

Urethral stricture disease treatment in low- and middle-income countries is fraught with challenges due to complex presentations and limited subspecialty training. Improper preoperative management, lack of specialty instruments, and suboptimal wound care all contribute to poor outcomes. International surgical groups like IVUmed who employ the “teach-the-teacher” model enhance local practitioner expertise and independence leading to long-term improvements in patient outcomes. Tailoring practice guidelines to the local resource framework and encouraging data collection and outcomes assessment are vital components of providing responsible care and should be encouraged.

**Electronic supplementary material:**

The online version of this article (10.1007/s00345-019-02819-2) contains supplementary material, which is available to authorized users.

## Introduction

As of 2010, the Global Burden of Disease (GBD) project estimated that benign urologic conditions accounted for 13.5 million disability-adjusted life years (DALYs) [1–3]. As the global burden of disease comprises a growing proportion of cases that require surgical care, the number of surgical mission trips has grown significantly [1]. Urethral strictures comprise one such disease that has been increasing in relation to trauma in low- and middle-income countries (LMICs) and requires dedicated urological care [4, 5]. The development of in-country permanent surgical centers in many parts of the world is leading to a paradigm shift where sustainability and local capacity building are increasingly more important than having visiting surgeons perform high-volume, short-duration surgical trips [6]. The task of focusing more on strengthening health systems is a daunting one made even more difficult by the lack of local health data and poor record collection systems in many LMICs [7]. As a result, urologic care in many parts of the world is still delivered in archaic forms that have historically high rates of morbidity and mortality [8]. While the most well-intentioned foreign surgeons attempt to address this surgical gap, the importance of data collection, outcome tracking, and quality of care has been neglected. We believe that the most sustainable approach to urethral stricture care and surgery in general in LMICs is a comprehensive one which includes:

- on-site data collection and documentation (regardless of a surgical trip);
- *teaching* as it relates to disease biology, preoperative evaluation, surgical principles, technical skills, and postoperative care;
- outcomes assessment for ongoing evaluation and quality improvement;
- cultural humility.

Without all of the above factors, a surgical program lacks accountability and is unlikely to lead to lasting improvements in care.

### Urethral stricture disease in LIMC

The risk factors for urethral stricture disease vary in high-income countries (HIC) and LMIC and can result in varying complexity of disease. In most HIC, urethral strictures are iatrogenic (39–45%) or idiopathic (30–36%) and only a small percentage is related to trauma or infection (11–15% and 4–6.5% respectively) [9–11]. Alternatively, data from West Africa show a much higher proportion of strictures caused by trauma and infection (up to 55% and 63% respectively) [12, 13]. The true prevalence and primary etiologies of stricture is unknown as most patients are initially seen in the community and published reports come from tertiary centers who are more likely to see recurrent or complex disease. It is well known that strictures related to trauma and infection can be quite technically challenging to repair. In HICs, complex strictures can be referred to dedicated reconstructive urologists. However, there are no urologic reconstructive training programs in West Africa. Patients who live outside of big cities often seek care with local providers who range from general practitioners to community health workers with no formal medical training and can result in significant disease-related morbidity.

### IVUmed

IVUmed is a US-based non-profit, urologic organization that has been working with international partners in over 30 cities worldwide since 1994. In contrast to most international surgical groups, IVUmed employs a ‘teach-the-teacher’ model. Their mission is to train local providers on-site to diagnose and manage complicated urologic diseases according to evidence-based principles that local providers can then impart to their own trainees. Historically, surgical workshops had been focused on teaching intra-operative technical skills to the local urologists. More recently, it has incorporated didactic portions to the workshops that focus on clinical knowledge and peri-operative management.

We explored the relationship between a large government hospital in Dakar, Senegal, and IVUmed to determine whether this surgical partnership led to any measurable changes in urethral stricture outcomes. We studied how urethral strictures presented to and were managed at this hospital over an 11-year period and assessed surgical outcomes. Our findings informed further intervention aimed at empowering the local urologists to practice evidence-based medicine and promote sustainable improvements in the treatment of urethral stricture disease.

## Method

This study employed both qualitative and quantitative methods, and was approved by the local hospital ethics board and the IRB at the University of Washington. The Hôpital General de Grand Yoff (HOGGY) in Dakar, Senegal is a major referral center in West Africa and was chosen as the site of study based on their 10-year relationship with IVUmed and hospital record keeping system which allowed for a retrospective review of data going back 11 years.

The relationship between IVUmed providers and the local urologists at the HOGGY was observed during a male reconstructive workshop in July 2017. This was the fifth dedicated male reconstructive workshop held since August 2013. Once the IVUmed team left, the host urologist was observed doing a variety of stricture repairs independently. Attention was paid to whether the teaching points from the workshop were being employed, and whether the technical skills employed during surgery seemed to have changed after the workshop.

Informal interviews were conducted with visiting urologists in attendance at the workshops to gain insight into the perceived value of the workshops and how or if they shape their practices. We did not include these qualitative results in our manuscript; however, it informed our understanding of how the program worked and helped contextualize our findings.

A retrospective review was performed on all urological surgical cases performed between January 1st 2006 and July 31st 2017 to evaluate the number and types of surgeries performed to treat urethral strictures. Medical records belonging to all patients who underwent urethroplasty were then reviewed for data extraction on demographics, etiology, prior treatments, stricture characteristics, diagnostic workup, surgical technique used, postoperative course, and whether or not IVUmed was involved. Outcomes were then compared before and after August 2013 when IVUmed’s reconstructive workshops began. Non-operative management was not assessed.

Surgical success was defined as a minimum of 6 months of follow-up over which there was no need for further intervention and the patient reported satisfaction with his urinary symptoms. The 6-month timeframe was used to determine success, because patients were not asked to return for follow-up if they were found to be doing well at their postoperative visit.

Failure was defined by patient report of difficulty urinating, recurrent stricture seen on cystoscopy, or posturethroplasty intervention.

### Inclusion criteria

All urethroplasty cases performed on men aged 19 or older between January 1st 2006 and January 31st 2017 were included for outcomes analysis. Patients with a history of prostate, bladder or urethral cancer were excluded from analysis. Two of IVUmed’s latest male reconstructive workshops were held within 6 months of this analysis, and as a result, the success of those urethroplasties could not be confirmed. However, if they failed within that timeframe, they were included in the analysis.

### Statistical analysis

Standard descriptive statistics for categorical and continuous variables were performed where appropriate. Continuous data are expressed as means with standard deviations. Categorical variables are shown as counts and its proportions. Data analysis was performed on total number of patients operated on and total number of urethroplasties performed. All categorical variables were compared using the χ^2^ test statistic and binary variables using the Student’s *t* test. Proportions of successful and failed operations among total cases performed were calculated by year. Procedures with unknown outcomes were excluded from the outcomes analysis. Logistic regression was used to estimate the odds of urethroplasty failure using both crude and multivariable models given IVUmed’s involvement, number and type of prior procedures performed, stricture length and location, interval between procedures, and surgical technique used. The odds of failure in the multivariable model were calculated relative to the date of IVUmed’s first male reconstructive workshop; that is, before and after 8/12/13. Statistical significance was set at *α* = 0.05. Statistical analyses were performed in Stata/SE version 14.2 (StataCorp LLC, College Station, TX, USA) and R version 3.5.0.

## Results

The workshop started with joint evaluation of all scheduled patients by the IVUmed provider and the staff urologist at the HOGGY. Their records were reviewed, and in any case with an inadequate preoperative workup, the IVUmed provider reviewed the necessary components of a complete and relevant workup or tailored the surgical plan accordingly. A total of 20 patients were seen and 7 urethroplasties were performed in 3 days. During surgery, the IVUmed provider guided the staff urologist where necessary and honed in on reconstructive principles that are not typically emphasized in general urology training. The dynamic between the two surgeons was collegial and mutually respectful. Notably, the IVUmed provider made a point of using only the available instruments, so that the skills and techniques being taught could be reproduced after the workshop.

**Fig. 1:**
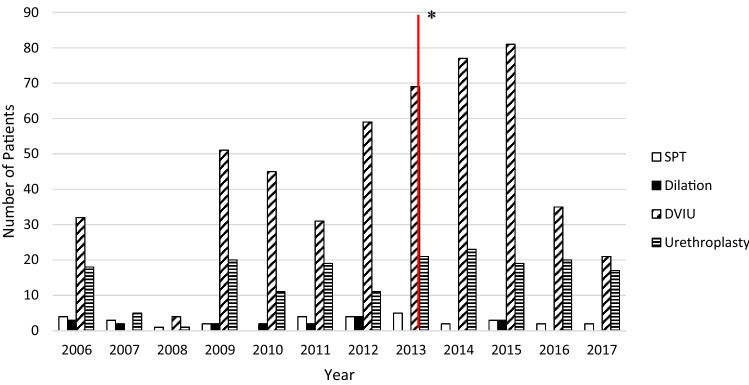
Surgical Management of Urethral Strictures at the HOGGY 2006–2017. Asterisk start of IVUmed male reconstructive workshops. *SPT* suprapubic tube, *DVIU* direct vision internal urethrotomy

Due to the absence of clinic records, the overall number of patients seeking care at the HOGGY for urethral strictures is unknown. Between January 1st 2006 and July 31st 2017, a total of 774 surgical procedures were performed to treat urethral strictures on 569 patients. Of these procedures, there were 539 direct vision internal urethrotomies (DVIU), 185 urethroplasties, 32 suprapubic tube placements (SPT), and 18 urethral dilations. Procedures performed in the ER and clinic were not captured. Figure 1 shows the types of surgical interventions performed by year.

Over the last 11 years, 115 patients met inclusion criteria for this study, and collectively, they underwent 145 urethroplasties. Table 1 shows demographic information on the 115 patients that were included in this study. Patients’ educational level and socioeconomic status were not noted in the medical records.

**Table 1:** Patient characteristics (*N* = 115)

Variable	*N* (%)
Age	
< 20	2 (1.7%)
20–39	42 (36.5%)
40–59	32 (27.8%)
60–79	36 (31.3%)
≥ 80	3 (2.6%)
Region of residence	
Dakar	82 (71.3%)
Other	33 (28.7%)
Documented comorbidities	
No PMH documented	52 (45.2%)
Diabetes mellitus	4 (3.5%)
Smoking	3 (2.6%)
Benign prostatic hyperplasia	11 (9.6%)
Bladder schistosomiasis	4 (3.5%)
Recurrent UTI/Urethritis	8 (7.0%)
Urolithiasis	1 (0.9%)
Renal insufficiency	3 (2.6%)
Other PMH	38 (33.0%)

*UTI* urinary tract infection, *PMH* past medical history

Most patients undergoing urethroplasty were young and otherwise healthy. The modal age at the time of surgery was 30. Many patients who presented to the HOGGY had been initially worked up elsewhere and, in most cases, had undergone some prior treatment for their stricture. Table 2 lists the proportion of patients who had undergone some prior surgical treatment for their strictures and describes the nature of the intervention.

**Table 2:** Prior management of stricture (*N* = 115)

Variable	Value
Documented prior surgical treatment of urethral stricture, *N* (%)	
One prior procedure	37 (32.2%)
Two or more prior procedures	47 (40.9%)
No prior procedure	31 (27.0%)
Documented prior dilation of urethral stricture, *N* (%)	
Yes	34 (30.9%)
No	76 (69.1%)
Unknown	5 (4.3%)
Documented prior DVIU, *N* (%)	
Yes	50 (45.5%)
No	60 (54.5%)
Unknown	5 (4.3%)
Documented prior urethroplasty, *N* (%)	
Yes	39 (33.9%)
No	71 (61.7%)
Unknown	5 (4.3%)

*DVIU* direct vision internal urethrotomy

Many of the patients who underwent urethroplasty at the HOGGY presented with long, complicated strictures. The most common documented causes of strictures were pelvic fracture urethral injury (PFUI) and infection (26.1% and 24.4%, respectively). Strictures caused by PFUI led primarily to bulbo-membranous strictures (28 of 30), of various lengths (40% < 2 cm, 23% > 2 cm, 37% undocumented length). In most cases, there was no etiology documented in the medical record. The vast majority of strictures were located in the distal urethra (82.6%). A smaller percentage was proximal (13%), and in 4.3% of cases, there was no documentation of stricture location and no accompanying X-rays that could be used to make the determination. Table 3 lists stricture characteristics documented in the medical records.

**Table 3:** Stricture characteristics at initial presentation (*N* = 115)

Variable	*N* (%)
Etiology of stricture	
PFUI	30 (26.1%)
Postinfectious (urethritis/GU soft-tissue infection)	28 (24.4%)
Iatrogenic (prior transurethral intervention)	25 (21.7%)
Unknown	32 (27.8%)
Location of stricture within urethra	
Proximal	15 (13%)
Distal	95 (82.6%)
Unknown	5 (4.3%)
Presenting symptoms	
Referred for known history of urethral stricture	41 (35.7%)
Difficulty urinating/complete retention	62 (53.9%)
GU soft-tissue infection	9 (7.8%)
Other	3 (2.6%)
Length of stricture (cm), *N* (%)	
≤ 2 cm	40 (34.8%)
> 2 cm	35 (30.4%)
Unknown	40 (34.8%)

*PFUI* pelvic fracture urethral injury, *GU* genitourinary

Excision and primary anastomosis (EPA) was the most common urethroplasty technique used (62% of urethroplasties). Seventeen percent of urethroplasties were performed using a flap or graft and the remaining 21% employed some other or unspecified technique.

### Effect of partnering with IVUmed

#### Increase in number and types of procedures performed

**Fig. 2:**
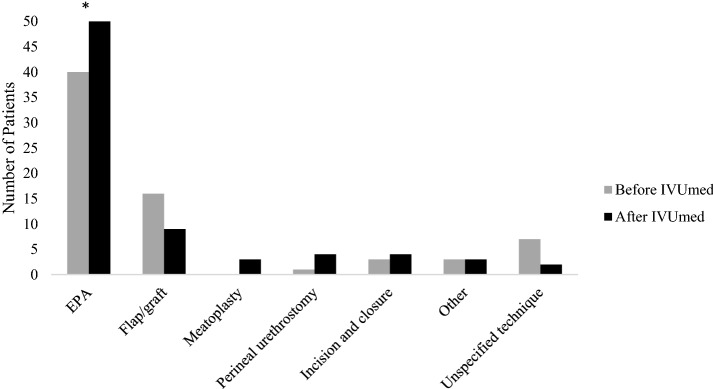
Urethroplasty techniques performed before and after IVUmed (*N* = 145). *EPA* excision and primary anastomosis. Asterisk number of EPA urethroplasties increased from a yearly average of 5.7–10 (*p* < 0.05)

The number and types of urethroplasty techniques used by the HOGGY urologists changed somewhat after IVUmed started its male reconstructive workshops in 2013. A total of 70 urethroplasties were performed on adults in the 7 years before IVUmed’s initiation of male reconstructive workshops (an average of 10/year). In the 4 years since then, 75 adult urethroplasties were performed (an average of 18.75/year). Seventeen of these were performed with IVUmed providers over the course of five workshops. There was a statistically significant increase in the yearly average of excision and primary anastomosis (EPA) urethroplasties from 5.7 to 10 (*p* value = 0.035) after the reconstructive workshops began. The yearly number of urethroplasties employing a flap or graft decreased slightly after the workshops began, but this was not statistically significant. In addition, meatoplasty as a technique was introduced after IVUmed began its workshops. The difference in the numbers of urethroplasties performed using other techniques was not statistically significant. Figure 2 shows the types of procedures performed before and after IVUmed’s reconstructive workshops began.

#### Improved success rates

There were 145 urethroplasties performed within the study period. A total of 19 urethroplasties were performed within 6 months of this analysis and 6 of those had failure documented in the chart. These 6 cases were included in the outcomes analysis. The remaining 13 cases were excluded from the analysis as their outcomes could not be confirmed. Of the 132 qualifying urethroplasties, outcomes were known on 118 who were included in the final outcomes analysis.

The outcomes after urethroplasties improved significantly after IVUmed’s workshops began in 2013. The success rate more than doubled from 12.7 to 29%, which leads to a reduction in the failure rate from 87.3 to 71% (*p* < 0.03).

The 14 patients lost to follow-up (7 before and 7 after the workshops) were compared and had no significant difference in any disease characteristics or types of procedures performed.

We took a more in-depth look at EPA urethroplasties, since these were the most common procedures performed before and after IVUmed’s involvement. A total of 73 EPA urethroplasties had known outcomes. Of these, 36 were performed before and 37 were performed after 8/12/13. The success rate for EPA urethroplasties doubled from 16.7 to 35.1% and failure rate in turn decreased from 83.3 to 64.9%, but this was not statistically significant (*p* = 0.07). The EPA technique accounted for the greatest proportion of successful cases (81%).

**Fig. 3:**
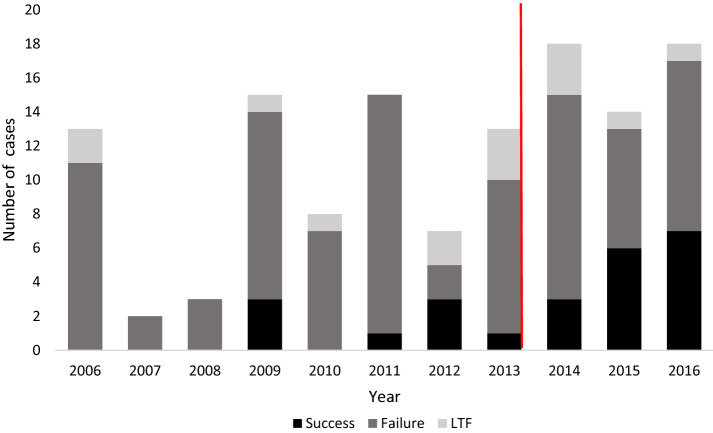
Urethroplasty outcomes by year. *LTF* lost to follow-up OR outcome too soon to be determined. Red line indicates start of IVUmed male reconstructive workshops

Of the 37 EPA urethroplasties performed after IVUmed’s involvement, 4 had documented involvement by IVUmed providers. One of these cases was successful, two-failed and one were lost to follow-up. Figure 3 shows the trend in urethroplasty outcomes after IVUmed started male reconstructive workshops (indicated by the red line). There were 19 cases performed in 2017 with less than 6 months of follow-up time, and among these, 6 had already presented as failures at the time of data collection. There were 13 cases whose outcomes were yet to be determined. There was no change in the success rate for procedures using a flap or graft. Success rates for unspecified techniques remained extremely low.

#### Factors associated with surgical outcome

The odds of failure for all urethroplasties were 65% lower after IVUmed’s workshops started in 2013, regardless of whether an IVUmed provider personally participated in the case or not (*p* = 0.05). Urethroplasties that were performed in the absence of any prior surgical intervention had 80% lower odds of failure (*p* < 0.01). A positive history of prior urethral dilation or DVIU was not associated with a statistically significant difference in outcomes. When the technique used was anything other than EPA or a graft/flap technique, the odds of failure were seven times higher (*p* = 0.05). Table 4 shows the odd ratios for procedure failure relative to several predictors.

**Table 4:** Factors associated with procedure failure (*N* = 118)

Predictor variables	Subgroup size (*N*)	OR of failure	95% CI	*p* value
Surgery performed after IVUmed reconstructive workshops				
No	118	Ref	Ref	
Yes, without IVUmed provider		0.38	0.14–0.99	0.05
Yes, with IVUmed provider		0.24	0.04–1.4	0.09
History of prior surgery				
No	118	Ref	Ref	
Yes, 1 prior		0.88	0.27–3.175	0.85
Yes, 2 or more		0.93	0.27–2.87	0.91
Type of prior surgery				
Urethral dilation	113	1.57	0.61–4.43	0.36
Prior DVIU	113	0.64	0.25–1.58	0.34
Time interval from last procedure to urethroplasty				
No prior procedure	79	Ref	Ref	
6 months or less		1.14	0.3–4.08	0.84
More than 6 months		0.86	0.24–2.88	0.81
Location of stricture				
Proximal	111	Ref	Ref	
Bulbar		0.43	0.06–1.76	0.3
Distal		0.74	0.1–4.0	0.74
Length of stricture	77			
Less than 2 cm		Ref	Ref	0.24
2 cm or longer		1.9	0.66–6.19	
Urethroplasty technique	117			
EPA		Ref	Ref	
Flap/graft		1.4	0.45–5.4	0.58
Unspecified/undescribed		8.1	1.55–150	0.05

*OR* odds ratio, *DVIU* direct vision internal urethrotomy, *EPA* excision and primary anastomosis

After removing procedures with missing data points on the variables of interest, we conducted a multivariable analysis on 110 cases. Notably, stricture length was excluded from the multivariable model due to the large number of missing data points. The odds of failure were 66% lower for all urethroplasties after IVUmed’s involvement when taking the covariates into account (*p* = 0.01). Importantly, this reduction in failure rate was sustained in cases that were performed without any IVUmed providers, but this was not statistically significant. The odds of failure were even lower if an IVUmed provider personally participated in the case, and this difference was statistically significant. The odds of failure were eight times higher when an unspecified technique was used (*p* = 0.05). Table 5 shows the results of the multivariable analysis.

**Table 5:** Factors associated with urethroplasty failure (*N* = 110)

Predictor variable	Odds ratio of failure	95% CI	*p* value
Procedure was done after IVUmed reconstructive workshops			
No	Ref	Ref	
Yes, without an IVUmed provider	0.37	0.11–1.08	0.08
Yes, with an IVUmed provider	0.12	0.02–0.87	0.03
Patient has had prior dilation of urethral stricture			
No	Ref	Ref	0.52
Yes	1.56	0.39–6.43	
Patient has had prior DVIU			
No	Ref	Ref	0.3
Yes	0.48	0.1–1.88	
Location of stricture			
Proximal	Ref	Ref	
Bulbar	0.41	0.06–1.9	0.3
Other	0.28	0.03–1.98	0.22
Urethroplasty technique			
End-to-end anastomosis	Ref	Ref	REF
Graft or flap procedure	1.33	0.3–6.9	0.71
Other	9.45	1.46–190	0.05

*DVIU* direct vision internal urethrotomy

#### Postoperative course

In the immediate postoperative period, 6.9% of all procedures were complicated by a surgical site infection (SSI). This rate remained unchanged before and after the workshops. The rate of wound breakdown (including fistula formation) decreased significantly, however, from 14.3 to 5.5%. The odds of wound breakdown or SSI were 10.4 times higher after a procedure involving a flap or graft (*p* < 0.01).

As the failure rates suggest, 81% of patients operated on before August 2013 required some additional intervention after urethroplasty. After the workshops, this fell to 55% (*p* < 0.01). Time to failure ranged from 1 to 23 months, but average was within 2 months. Postoperative interventions for failed procedure included repeat transurethral procedures (dilations and DVIUs), repeat urethroplasties, and urethral fistula repairs.

The typical length of postoperative follow-up was 2 months. However, the total length of follow-up ranged from 1 to 127 months with the longer time frame consisting mainly of patients who had difficulty urinating and required multiple additional interventions.

## Discussion

The approach to international partnerships that focuses more on training and less on case volumes is relatively new in the global health landscape. Few organizations track or present their data. While there is clearly room for improvement, the fact that urethroplasty outcomes improved after training workshops is all the more reassuring given the small number of procedures that were actually performed by IVUmed providers. Because of the 6 months of follow-up needed to determine success, the cases included in the outcomes analysis encompassed only 3 IVUmed workshops. As there has been no change in the complexity of disease presenting to the HOGGY or the mismanagement by referring providers, the decrease in failure rates suggests that the surgical decision-making and technical skills taught during the workshops may gradually be manifesting through better patient outcomes. As the number of workshops increases and providers gain more experience, we hope to see continued improvement.

These data support the argument that teaching local providers about the principles of reconstructive urology has a more sustainable impact on patient outcomes than simply conducting a high-volume surgical trip. Having said that, the failure rates are still unacceptably high and there are many different factors that may be responsible for this in and outside of the operating room.

We believe, where possible, that it is best to have a dedicated local urologist engaged in multiple workshops until he or she is comfortable not only performing the procedure independently but teaching those techniques to their colleagues and trainees. As a result of this discussion, a provider from the HOGGY has been assigned to assume the role of reconstructionist and will be participating in all subsequent workshops.

There were multiple occasions on which a patient was found to have a worse stricture intra-operatively than was anticipated based on improperly performed X-rays. This sometimes led to an inappropriate surgical technique being applied to certain strictures or surgeons having to perform much more technically challenging procedures than they were prepared for. Based on this experience, the local urologist arranged for a training session for radiologists on how to properly perform the diagnostic X-rays needed for urethral strictures.

We also discussed several non-operative issues that arose from making observations during surgery. Sterile technique was not strictly followed. This was due in part to the paucity and expense of certain ‘single-use’ instruments which were reused multiple times and could not be fully sterilized. Many of the surgical instruments were in poor condition and affect tissue handling adversely.

Postoperative care for urethroplasties requires diligent wound care and the HOGGY did not have specialized wound care supplies for urethroplasties that employ grafts. This certainly could have contributed to the poor success rate of those procedures and the SSI rate. Due to the nature of the health care system and the lack of resources in general, patients are not closely attended to by nursing staff and there are few protections for fresh surgical wounds. This increases the risk of postoperative tissue trauma which can seriously hinder wound healing.

Other factors identified during the study that could contribute to poor patient outcomes and are difficult to address within our framework include late presentation by patients who live in rural areas, mismanagement elsewhere, and high rates of urethritis causing long and complex strictures.

### Additional intervention

Perhaps most importantly, we collaborated with the local urologist to create a set of management guidelines for urethral strictures that can guide providers on the proper diagnostic workup and selecting the most appropriate surgical technique for different strictures. These guidelines were drawn from the American Urologic Association’s recommendations [13] which are based on the best available evidence. Because the nature of urethral strictures and the resources available at the HOGGY are quite different from what is typically seen in the US, the guidelines were modified and are included in the Online Appendix. We hope that the guidelines will not only prevent avoidable errors in management but also promote a standard of care that all patients can benefit from. This final concept is important in all low-resource surgical settings where management algorithms do not exist and providers have various levels of expertise.

### Limitations

The lack of national data on the prevalence of urethral strictures, management practices, and outcomes from urethral stricture management created a lack of context within which to assess the HOGGY’s data. In addition, we were unable to judge the size of the population that sought care for urethral strictures at the HOGGY, because we did not have ER and clinic records. As a result, we could not assess non-operative management. Another major limitation was the lack of routine follow-up beyond the immediate postoperative period which required shortening our timeline for success to 6 months. As a result, the procedures performed in more recent workshops were included only if they had evidence of failure, but could not be included in the proportion of successful cases, which they may ultimately comprise. Several patients had unknown outcomes and their exclusion lowered the power of statistical analysis.

The medical records were hand-written in Senegalese French and at times, pertinent records were either missing or damaged. Deciphering hand-writing and translating between languages may have introduced errors in transcription. Operative notes were often lacking in detail and did not always specify surgical technique or stricture characteristics. In particular, stricture length was not accounted for on multivariable analysis because of too many missing data points. However, the univariate analysis showed that the outcome of the procedure after IVUmed’s involvement was not related to stricture length.

There was no way to quantify surgeon confidence in performing different urethroplasty techniques before and after the workshops. The finding that EPA urethroplasties increased after the workshops might act as a surrogate measure of confidence, but further study would be required to make this conclusion. Further investigation of surgeon confidence in the various techniques is warranted, and future IVUmed trainings may consider standard assessments of surgeon skills and confidence before and after workshops.

## Conclusion

Outcomes for urethroplasty improved significantly after only three surgical workshops that were aimed at improving not only technical skills but knowledge of surgical principles and peri-operative management. In attempting to assess outcomes before and after dedicated training sessions, we aim to take a responsible approach to global health and hope to encourage other groups to do the same. International surgical organizations should focus on empowering local providers and promoting the independent practice of safe surgical care rather than fostering a reliance on visiting surgeons. Creating a set of management guidelines in collaboration with local providers can be an invaluable step forward in this process.

## Electronic supplementary material

Below is the link to the electronic supplementary material.

Supplementary material 1 (DOCX 15 kb)
