# Supplementary material for: The role of international partnerships in improving urethral reconstruction in low- and middle-income countries
Source: World J Urol. 2019 Jun 8;38(12):3003–11. doi: 10.1007/s00345-019-02819-2 (PMC7716901; doi:10.1007/s00345-019-02819-2)
Supplement: Supplementary file 1 — Supplementary material 1 (DOCX 15 kb) [file 345_2019_2819_MOESM1_ESM.docx]

APPENDIX

**Management of Urethral Strictures – derived from AUA guidelines on Urethral Strictures 2016 and tailored to the HOGGY.**

**Initial Diagnosis and Workup:**

1. The differential diagnosis of any patient presenting with weak force of stream, incomplete emptying, pain with voiding, recurrent UTIs and rising post-void residual should include urethral stricture.
2. The initial workup of all patients should include a history and physical exam, urinalysis, urine flow parameters, post void residual and assessment of voiding symptoms using a validated questionnaire.
3. Cystoscopy or retrograde urethrogram/voiding cystourethrogram may be used to confirm the diagnosis of a suspected stricture.
4. For strictures requiring urgent treatment (in the event of retention), physicians may place a suprapubic tube, dilate or perform a direct vision internal urethrotomy (DVIU).
5. Strictures that can be managed non-urgently MUST be evaluated to determine length and location. This can be accomplished with a combination of retrograde urethrogram and voiding cystourethrogram to fully evaluate the urethra proximal and distal to the point of narrowing.

**Management of Bulbar Strictures measuring less than 2cm in length:**

1. First-time treatment may consist of dilation, DVIU or urethroplasty
2. Foley catheter should be removed 72 hours after an uncomplicated dilation or DVIU
3. If the patient is not a candidate for urethroplasty, they may be managed with long-term self-catheterization after DIVU
4. Recurrent anterior strictures after failed dilation or DVIU should be offered urethroplasty instead of repeated transurethral procedures due to the diminishing success rate.
5. Surgeons who do not perform urethroplasties should refer their patients to those with expertise.

**Management of Bulbar Strictures measuring 2cm in length or longer:**

1. Surgeons should offer urethroplasty as the initial management for long bulbar strictures given the low success rate of dilation and DVIU.
2. Strictures measuring 2 or more centimeters should be reconstructed with the use of grafts/flaps.
3. Excision and end-to-end anastomosis should not be performed due to the risk of penile shortening and curvature.

**Management of Anterior Strictures:**

1. First-time urethral strictures of the meatus or fossa navicularis can be treated with dilation or meatotomy.
2. Recurrent meatal or fossa strictures should be offered meatotomy.
3. Patients with pendulous penile urethral strictures should be offered urethroplasty because of the high recurrence rate with endoscopic procedures.

**Long, multi-segment strictures:**

1. Reconstruction can be performed in 1 stage or multiple stages using grafts or pedicled flaps. Buccal mucosa is the preferred tissue for grafting. Pedicled flaps should not involve hair-bearing skin.
2. Perineal urethrostomy is an alternative long-term treatment option to urethroplasty.

**Pelvic fracture Urethral Injury (PFUI):**

1. PFUI should not be treated endoscopically; instead, delayed urethroplasty should be performed.

**Bladder neck contracture:**

1. Bladder neck contracture after endoscopic prostate procedures or open prostatectomy may be treated with dilation, transurethral incision (TUIBN), or transurethral resection (TURP).
2. Open reconstruction may be performed for recurrent bladder neck contractures.

**Intra-operative guidelines for urethroplasty:**

1. ALL instruments and equipment on the operative field, must be sterile.
2. The urethral mucosa should be handled with fine, toothed forceps to prevent any ‘crush injury’ that could promote re-stricturing.
3. The bulbospongiousus should be handled with broad, non-toothed forceps to prevent bleeding.
4. The urethra should be mobilized 2-3cm proximally and distally to the area of the stricture to allow a tension-free anastomosis.
5. The anastomosis of the urethral mucosal edges should be ‘water-tight’ to prevent fistula formation.
6. Urethral strictures must be excised or augmented to prevent recurrence. Urethrotomy alone is insufficient.

**Urethral biopsy:**

1. Biopsy may be performed for suspected lichen sclerosis/BXO, and must be performed for suspected cancer involving the urethra.

**Post-operative follow up:**

1. Patients should be maintained on oral antibiotics based on the hospital’s antibiogram for the duration of urethral catheterization.
2. The urethral should be re-evaluated with a retrograde urethrogram/voiding cystourethrogram 2-3 weeks after surgery on catheter removal. If there is evidence of a leak, the catheter should be replaced.
3. Clinicians should monitor urethral stricture patients with regularly scheduled surveillance visits to identify symptomatic recurrence up to at least 2 years from the time of surgery.
